# Sedentary behavior in children, adolescents, and young adults treated for cancer: A scoping review of health outcomes and interventions

**DOI:** 10.1007/s11764-025-01908-z

**Published:** 2025-11-11

**Authors:** Brittany J. Ivory, Kristen Moore, Jennifer E. Dinalo, Nina S. Kadan-Lottick, Christina M. Dieli-Conwright, Etan Orgel, Kirsten K. Ness, David R. Freyer, Britni R. Belcher

**Affiliations:** 1 Cancer and Blood Disease Institute, Children’s Hospital Los Angeles, 4650 Sunset Blvd, MS #54, Los Angeles, CA 90027 USA; 2 Department of Pediatrics, Keck School of Medicine, University of Southern California, Los Angeles, CA USA; 3 Department of Population and Public Health Sciences, Keck School of Medicine, University of Southern California, Los Angeles, CA USA; 4 Southern California Evidence Review Center, Keck School of Medicine, University of Southern California, Los Angeles, CA USA; 5 Department of Oncology, Lombardi Comprehensive Cancer Center, Georgetown University Medical Center, Washington, DC USA; 6 Division of Population Sciences, Dana-Farber Cancer Institute and Harvard Medical School, Boston, MA USA; 7 Department of Epidemiology and Cancer Control, St. Jude Children’s Research Hospital, Memphis, TN USA; 8 Department of Medicine, Keck School of Medicine, University of Southern California, Los Angeles, CA USA

**Keywords:** Childhood cancer, Adolescent and young adult (AYA) cancer, Cancer survivors, Sedentary behavior, Health outcomes, Behavioral interventions

## Abstract

**Purpose:**

Although sedentary behavior (SB) is linked to adverse health outcomes in adult cancer populations, its health impact and the effectiveness of SB interventions in younger patients remain underexplored. This scoping review examined (1) associations between SB and health outcomes and (2) intervention studies to reduce SB among childhood and AYA cancer patients and survivors.

**Methods:**

A search of five databases (PubMed, Embase, Web of Science, CINAHL, SportDiscus) was finalized in May 2025. Studies were included if ≥ 50% of participants were ≤ 39 years old and treated for cancer, and SB was measured via device or self-report. Observational studies had to report associations of SB with health outcomes; intervention studies had to report results for SB-related aims. Risk of bias and quality assessments were performed using established tools.

**Results:**

Twenty-four studies (16 observational, 8 interventional) met criteria. Observational studies identified associations between SB and adverse cardiometabolic, bone, and patient-reported outcomes; none assessed mortality. All interventions primarily targeted physical activity, not SB reduction; three showed modest, short-term SB decreases.

**Conclusions:**

Early evidence suggests SB is associated with multiple adverse health outcomes in youth treated for cancer, while current interventions without an SB-specific focus have minimal impact. Key research gaps include the absence of longitudinal data, standardized SB measurement, and randomized controlled trials testing SB-focused strategies.

**Implications for Cancer Survivors:**

Reducing SB may help mitigate health risks in childhood and AYA cancer survivors. More robust epidemiologic evidence and targeted interventions are needed to address this modifiable behavior.

**Supplementary Information:**

The online version contains supplementary material available at https://doi.org/10.1007/s11764-025-01908-z.

## Introduction

Due to advances in treatment, 5-year overall survival now exceeds 86% among children and adolescents and young adults (AYAs; ages 15–39 years) with cancer [1]. Consequently, there is a growing population of long-term survivors at increased risk for developing late effects that contribute to excess morbidity and premature mortality [2–4]. Importantly, these include chronic health conditions such as obesity, impaired glucose metabolism, reduced bone mineral density, and cardiovascular disease [2–7], which have been linked to physical inactivity and excessive sedentary behavior (SB) in the general population [8–11].

SB is defined as any waking activity with energy expenditure ≤ 1.5 metabolic equivalents (METs) while sitting or lying [12]. SB is distinct from physical inactivity, which refers to insufficient moderate to vigorous physical activity (MVPA) [12, 13]. SB and MVPA are only weakly correlated [12, 13], and high levels of SB have been associated with increased risk for morbidity and mortality across multiple adult populations independent of MVPA levels [8, 10, 12, 13], suggesting SB is a distinct behavioral risk factor. Among childhood and AYA cancer patients, SB becomes highly prevalent early during treatment, comprising up to 80% of waking hours [14], and often persists throughout and beyond therapy, leading to long-term sedentary lifestyles [14, 15].

Negative health effects of SB have been increasingly recognized in adult cancer patients and survivors, with SB associated with excess all-cause and cancer-specific mortality [16–19]. In addition, SB has been linked with worse patient-reported outcomes (PROs) among adults with cancer, including increased fatigue, pain, depressive symptoms, and lower quality of life [20–23]. In contrast, little is known about the effects of SB in childhood and AYA cancer patients and survivors, who often live for many decades after treatment.

Targeted interventions to reduce SB may offer an effective strategy for mitigating health risks and improving health trajectories in this population. Particularly during active treatment, reducing SB by interrupting sedentary time with light activity throughout the day may be more achievable than increasing MVPA, as it typically requires no special equipment or training and may require less physical exertion. However, existing literature in childhood and AYA oncology has been focused primarily on MVPA rather than SB. In 2022, two authors from our team (BRB and CDC) published a systematic review of SB interventions that revealed a general trend toward reduced SB in intervention groups, but it was limited to adult cancer patients and survivors [24]. Collectively, the high prevalence of SB in youth with cancer, the predominance of MVPA-focused studies in pediatric oncology, and the limitation of SB intervention evidence to adult populations reveal a critical gap that warrants synthesis of existing literature in childhood and AYA oncology.

To address this gap, this scoping review was undertaken to (1) summarize existing observational studies on the association between SB and health outcomes, including cardiometabolic risk, patient-reported outcomes, morbidity, and mortality in childhood and AYA cancer patients and survivors; and (2) describe the results of SB intervention trials conducted in this population. Our overall objective was to evaluate current knowledge about SB in pediatric and AYA oncology to inform future research and intervention development.

## Methods

Methodological details for this review are previously published [25]. The scoping review is registered through Open Science Framework (OSF) (https://osf.io/7apyv) and is reported according to the Preferred Reporting Items for Systematic Reviews and Meta-Analyses extension for Scoping Reviews (PRISMA-ScR) [26]. In March 2025, we searched the PubMed, Embase, Web of Science, CINAHL and SportDiscus databases (updated in May 2025). The search strategy was developed by the first and second authors (BJI and KM) and finalized by an experienced medical librarian (JD). Medical Subject Heading (MeSH) terms, their relevant synonyms, and the Boolean operators “AND” and “OR” were used to build the search strategy based on three concept clusters: (1) pediatrics, (2) cancer patients and survivors, and (3) sedentary behavior. The search strategy was run by JD. The full electronic search strategy for PubMed is shown in Supplemental Table 1 and in the study protocol [25].

The Covidence systematic review software (Verita Health Innovation, Melbourne, Australia; www.covidence.org) was used to manage and screen the references. References were extracted as text files and imported into Covidence. Duplicates were removed using Covidence, and if any were missed, they were removed manually. Titles, abstracts, and full texts were screened by two independent reviewers (BJI and KM) in Covidence. Reference lists of review articles were reviewed by BJI and KM for additional relevant studies and imported into Covidence. Discrepancies were discussed at each stage and resolved by consensus. Full texts were obtained for all articles identified as eligible by both reviewers, with final expert review by BRB.

Study eligibility criteria have been previously described [25]. Key inclusion criteria were (1) published in English in a peer-reviewed journal after January 1, 2000; (2) participants currently or previously treated for any type of cancer; (3) 50% or more of participants age 39 years or less at the time of study; and (4) measurement of SB via device or self-report. All original study designs were eligible. Observational studies needed to report on the association of SB with a specific health outcome. Intervention studies needed to include SB reduction as a primary, secondary, or exploratory aim and report change in SB.

Data extraction was completed in June 2025 using Covidence (BJI and KM). The data extraction template was built in Covidence by the first author (BJI). For all studies, data were extracted for first authors(s); study year(s), country, design, and duration; sample size; demographics; cancer types and treatment status; SB measures; and relevant covariates. For observational studies, data on the relationship between SB and any reported health outcomes were also recorded; for intervention studies, intervention type, frequency, duration, adherence and outcomes were extracted. Results for the entire sample, within-group differences, and/or between-group differences were reported where applicable. A *p*-value < 0.05 was considered statistically significant.

Study quality assessment was conducted by two independent reviewers (BJI and KM) with final expert review by BRB. For observational studies, methodological quality was evaluated using the NIH Quality Assessment Tools for Observational Cohort and Cross-Sectional Studies or for Case–Control Studies, as appropriate [27]. To assess internal validity, the Cochrane Risk-of-Bias Tools for Randomized Trials – Version 2 (ROB 2) [28] was used for randomized controlled trials (RCTs) and the Cochrane Risk of Bias In Non-randomized Studies of Interventions (ROBINS-I) [29] for non-randomized intervention studies. Discrepancies were adjudicated and consensus reached for all included studies.

## Results

### Study Selection

**Fig. 1:**
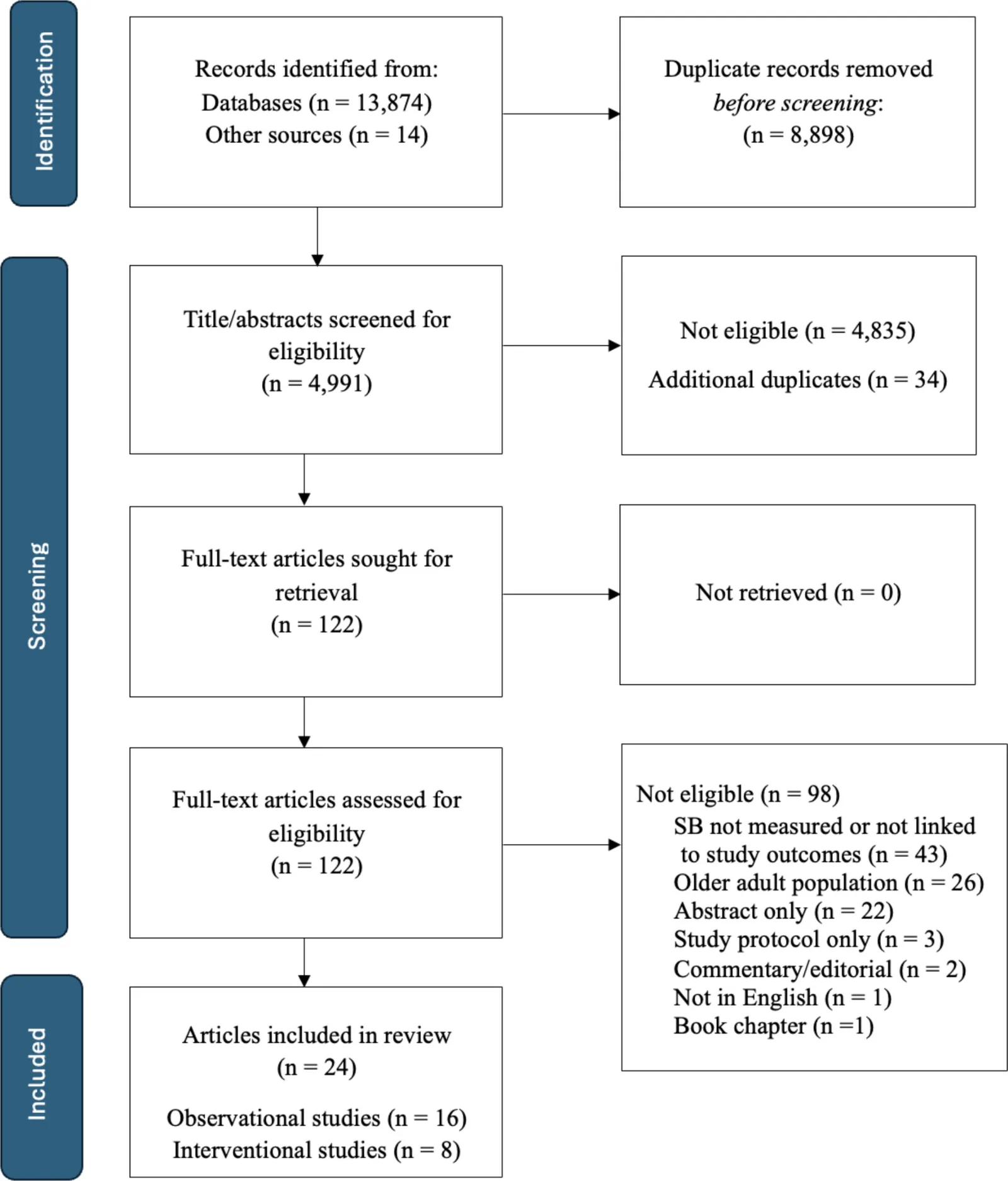
PRISMA flow diagram

A total of 13,784 records were identified through database searches, and 14 articles were added from reference lists review. After removal of duplicates (*n* = 8,989), title/abstract screening was completed for 4,991 records, yielding 122 records for full text review. After the full-text review, 24 manuscripts were included in this review (Fig. 1). Among the 24 included articles, 16 were observational [14, 30–44] and 8 interventional [45–52].

Although the National Cancer Institute defines AYAs as individuals 15–39 years of age [53], nearly all included studies used age 18 years to distinguish between childhood/adolescent and adult populations. Many studies focused on populations either on or off treatment; some included both. Thus, consistent with our populations of interest, this review encompasses four patient groups: (1) child/adolescent patients on treatment (age at diagnosis and enrollment < 18 years; on therapy); (2) child/adolescent survivors of childhood/adolescent cancer (age at diagnosis and enrollment < 18 years; off therapy); (3) young adult (YA) cancer patients on treatment (age at diagnosis and enrollment 18–39 years; on therapy); and (4) YA/adult survivors of childhood/adolescent or YA cancer (age at diagnosis ≤ 39 years, age at enrollment ≥ 18 years). The population of survivors enrolled at > 39 years of age was very small (estimated < 50 survivors).

### Associations between SB and Health Outcomes

#### Study Characteristics

Included observational studies are summarized in Table 1. Fourteen of the 16 observational studies (87.5%) were cross-sectional [14, 31–36, 38–44], one was a case–control study [37], and one was a prospective study with median follow-up of 5.2 years [30]. Publication dates ranged from 2009 to 2024, with most (*n* = 10) studies published since 2015 [14, 30, 31, 33–35, 38, 41, 42, 44]. The studies were conducted in the United States (U.S.) (*n* = 5) [30, 32, 36, 40, 43], Spain (*n* = 4) [34, 38, 41, 42], Poland (*n* = 2) [31, 44], and 1 each for Australia [33], the Netherlands [14], Norway [37], and Turkey [39]; one included 6 international sites [35].

**Table 1:** Details of included observational studies

Author(s), year published, country	Study design	Inclusion criteria	Study aims	Sample size and characteristics	Cancer diagnoses	Treatment history	SB measurement and results	Main outcomes	Quality Rating
*Studies reporting on cardiometabolic outcomes*									
Miller et al., 2010^32^ USA	Cross-sectional	Age at diagnosis: NR (≥ 3 yrs from diagnosis) Age at enrollment: Any age Treatment status: Off therapy	Compare measures of adiposity and related metabolic factors in childhood cancer survivors vs. siblings Identify treatment, demographic, lifestyle, and endocrine factors associated with adiposity in survivors	Total *N* = 241 _______________ Survivors: *n* = 170 Mean age: 19.4 yrs Range: 5.9–39.7 yrs Males: 87 (51%) Females: 83 (49%) White: 100% Non-Hispanic: 100% ________________ Sibling controls: *n* = 71	All cancer types Mean age at diagnosis: 7.5 yrs Range: 0–24.1 yrs	Off therapy: 100% Mean years since diagnosis: 11.9 yrs Range: 4.3–31.6 yrs ___________________ Treatment received: CT: ≥ 66% (Exact n not reported) Cranial RT: 81 (48%)	Measures: Self-report—Adapted questionnaire based on prior NHANES questions related to SB: time spent watching television per day (hrs/day) Results: Male survivors TV time greater than siblings: 2.3 vs. 1.8 h/day* No significant difference in TV time for female survivors vs. siblings: 2.2 vs. 2.1 h/day	Outcome type and measurement: Body composition: BMI (kg/m2), total body fat and total trunk fat measured by DEXA Results: In survivors: TV viewing time not associated with BMI in multivariate models ↑ TV viewing time associated with ↑ body fat and ↑ trunk fat*	Good
Braam et al., 2016^14^ The Nether-lands	Cross-sectional	Age at diagnosis: < 18 yrs Age at enrollment: 8–18 yrs Treatment status: On therapy or ≤ 1 yr post	Assess CRF during or shortly after treatment Evaluate association of CRF with PA and SB	Total N = 60 Mean age (SD): 12.6 yrs (3.1) Males: 35 (58%) Females: 25 (42%) _________________ Controls: NA	All cancer types	On therapy: 17 (28%) Off therapy: 43 (72%) __________________ Chemotherapy alone (CT): 37 (62%) CT + radiation therapy (RT): 7 (18%) CT + surgery (SG): 8 (13%) CT + RT + SG: 8 (13%)	Measurement: Device—Actical accelerometer (worn on hip for 4 days during waking hours): total ST (min/day) and sedentary bouts Results: Total ST (min/day) Median (IQR): 869 min/day (785–911) 76% of waking hours Daily sedentary bouts Median bouts ≥ 30 min (IQR): 4 (3–6) Median bouts ≥ 60 min (IQR): 1 (0–2)	Outcome type and measurement: CRF: VO2_peak_ via CPET (ml/kg/min) Results: ↑ Total ST (min/day) associated with ↓VO2_peak_. Every additional min of ST/day ↓VO2_peak_ by 0.06 ml/kg/min.*	Fair
Howell et al., 2018^30^ USA	Prospective cohort study	Age at diagnosis: NR (≥ 10 yrs from diagnosis of childhood cancer) Age at enrollment: ≥ 18 years Treatment status: Off therapy	Describe SB and PA behaviors in survivors vs. controls Cross-sectionally examine association between SB and body composition outcomes Prospectively examine association between SB and new onset (or increased severity) of cardiovascular disease (CVD) risk factors in survivors	Total *N* = 661 _________________ Survivors: *n* = 330 Mean age (SD): 28.9 (6.1) Males: 172 (52%) Females: 58 (48%) White: 289 (88%) Non-Hispanic: 316 (96%) Mean follow-up: 5.2 yrs _________________ Matched community controls: *n* = 331	ALL Median age at diagnosis: 5.1 yrs Range: 0.7–18.8 yrs	Off therapy: 100% ___________________ Chemotherapy: 100%	Measurement: Device—ActiGraph wGT3x-BT accelerometer (worn for 7 days): Total ST (min/day) Sedentary defined as ≥ US population mean of 60% of day spent in ST Self-report – Leisure screen time via 1 question on daily time sitting and watching TV or using a computer outside of work over past 30 days (hrs/day) Results: Survivors similarly sedentary to community controls Mean % sedentary/day: Survivors: 65% Controls: 67% Number defined as sedentary: Survivors: 235 (71%) Controls: 257 (78%) Mean total ST (min/day) Survivors: 449.1 min/day Controls: 473.4 min/day Screen time ≥ 5 h: Survivors: 83 (25%) Controls: 61 (18%)	Outcome type and measurement: Body composition: BMI, weight-to-height ratio (WtHR), lean mass measured via DXA CVD risk factors included hypertension, high cholesterol, hypertriglyceridemia, obesity, and abnormal glucose metabolism. Obtained from clinical and lab testing. Graded according to CTCAE version 4.03 Results: In survivors: ↑ Total ST (min/day) associated with ↓ lean mass* ≥ 5 h/day leisure screen time associated with ↑ WtHR and ↓ lean mass vs ≤ 2 h/day* Being sedentary (≥ 60% day) associated with ↑ risk of new onset high total cholesterol and any CVD risk factor*	Good
Kowaluk and Woźniewski, 2020^31^ Poland	Cross-sectional	Age at diagnosis: NR Age at enrollment: NR Treatment status: On therapy	Assess peak oxygen (VO2_peak_) uptake in children undergoing leukemia treatment vs. expected values in healthy children Assess the influence of treatment methods and SB on the level of exercise capacity in children with leukemia	Total *N* = 21 Mean age (SD): 10.7 yrs (2.0) Range: 7–13 yrs Males: 12 (57%) Females: 9 (42%) _________________ Controls: NA	ALL: 13 (62%) AML: 8 (38%)	On therapy: 100% Mean treatment duration (SD): 6.2 mos (1.6) _____________________ CT: 100%, receiving in hospital	Measure: Self-report – Health Behavior in School-Aged children (HBSC 2018) questionnaire: 4 questions on time in front of screens, playing sedentary games, and using the Internet over the last 7 days (each in hrs/day) Results: % [of participants] with ≥ 5 h/day screen time: 95% on weekdays 100% on weekends % playing sedentary games daily: 100% Time on weekends > weekdays % reporting Internet use for ≥ 4 h/day: 100%	Outcome type and measurement: CRF: VO2_peak_ (ml/kg/min) via CPET Results: No significant effect of increased SB in any domain (screen time, video games, internet) on VO2_peak_	Poor
Mizrahi et al., 2020^33^ Australia	Cross-sectional	Age at diagnosis: NR Age at enrollment: 8–18 yrs Treatment status: Off therapy ≥ 1 yr	Measure self-reported PA and fitness levels in survivors Identify predictors of fitness and physical activity stage of change in survivors	Total *N* = 102 Mean age (SD): 12.8 (3.3) yrs Males: 63 (62%) Females: 39 (38%) _________________ Controls: NA	All cancer types Mean age at diagnosis (SD): 5.4 (4.6) yrs Range: 0–16 yrs	Off therapy: 100% Mean time since therapy completion (SD): 6.0 (4.0) yrs Range: 1–14.5 yrs _____________________ Treatment received: CT: 100 (98%) SG: 72 (71%) RT: 27 (27%) BMT: 20 (20%)	Measures: Self-report—Questionnaire for screen time: hours per school day and weekend day spent (a) watching television, (b) playing video games or (c) using a tablet/mobile phone for non-educational purposes; summed for total screen time (hrs/day) Results: Mean total daily screen time (SD): 5.54 (2.89) hrs Time on weekend days > week days	Outcome type and measurement: CRF: VO2_max_ (ml/kg/min) via 6-min walk test (6MWT) using published algorithm in CCS Results: ↑ screen time (hrs/day) associated with ↓ VO2_max_ ↑ screen time showed more negative effect on fitness when PA ↓ *	Good
*Studies reporting on patient-reported outcomes (PROs)*									
Orsey et al., 2013^36^ USA	Cross-sectional	Age at diagnosis: < 18 yrs Age at enrollment: 8–18 yrs Treatment status: On therapy	Investigate relationship between PA and sleep in outpatient pediatric cancer patients undergoing treatment	Total *N* = 36 Children (8–12 yrs): 23 (64%) Adolescents (13–18 yrs): 13 (36%) Males: 26 (72%) Females: 10 (29%) White: 24 (67%) Hispanic: 4 (11%) __________________ Controls: NA	All cancer types	On therapy: 100% _____________________ Treatment: CT: 36 (100%) RT: 11 (31%)	Measurement: Device—Actiwatch 2 accelerometer (worn on wrist for 7 days): immobile time (min/day) Results: Immobile time accumulated in survivors not reported	Outcome type and measurement: Sleep measured objectively via Actiwatch 2 and self-report measures recorded in sleep diaries Results: ↑ immobile time (min/day) associated with ↓ sleep efficiency, ↑ wake after sleep onset, ↓ # of wake bouts, ↓ sleep time* No association with self-reported sleep quality. ↑ immobile time associated with ↑ morning mood*	Good
Zeller et al., 2014^37^ Norway	Case–control study	Age at diagnosis: NR (≥ 5 yrs from diagnosis of childhood cancer) Age at enrollment: ≥ 18 yr Treatment status: Off therapy	Assess persistence of CF (PCF) in long-term childhood cancer survivors Compare survivors with PCF to those without CF regarding associated factors, including PA and SB	Total N = 62 Median interval between fatigue assessments (IQR): 2.7 yrs (1-4.3 yrs) _______________ Cases (PCF): 27 Mean age (SD): 33.7 (6.6) yrs Female: 18 (67%) ________________ Controls (no PCF): 35 Mean age (SD): 34.4 (7.3) yrs Female: 19 (54%)	ALL and lymphoma Median age at diagnosis: 10.1 yrs Range: 1.6—18.4 yrs	Off therapy: 100% ____________________ Cases: Mean off therapy follow-up time (SD): 23.0 (8.2) yrs CT: 100% RT: 13 (43%) ____________________ Controls: Mean off therapy follow-up time (SD): 24.0 (7.9) yrs CT: 100% RT: 14 (57%)	Measurement: Device – ActivPAL accelerometer (worn for 7 days): time spent supine/stitting (total ST, hrs/day); time standing (hrs/day), steps/day, and # sit/stand transitions/day Results: Survivors with PCF similarly sedentary to controls Mean total ST (SD): PCF: 18.5 (1.8) hrs/day Controls: 17.6 (SD 1.9) hrs/day	Measurement: Device-measured SB assessed as a predictor of PCF Results: No significant associations reported between total ST (hrs/day) and PCF Survivors with PCF had ↓ stepping time and steps.*	Fair
Collins and McGowan, 2018^35^ Canada, USA, UK, Ireland, Australia and New Zealand	Cross-sectional	Age at diagnosis: ≥ 18 yrs Age at enrollment: 18–44 yrs Treatment status: On and off therapy	Measure and explore relationships of domain-specific SB, levels of PA, and QoL in young adult cancer survivors	Total N = 96 [*Baseline N for demographics* = *147*] Mean age (SD): 31.9 (5.7) Males: 26 (18%) Females: 120 (82%) Other: 1 (< 1%) White: 131 (89%) Other: 16 (11%) _________________ Controls: NA	All cancer types Cancer status Disease free: 65 (68%) Current disease: 16 (17%)	Off therapy: 72 (75%) On therapy: 24 (25%) ____________________ Treatment: SG: 69 (72%) RT: 46 (48%) Hormone therapy: 21 (22%) Chemotherapy: 68 (71%)	Measures: Self-report—SIT-Q: Measured total and domain-specific SB (sleeping/napping, meals, transportation, work, child and elder care, leisure activities) in hrs/day on weekends and week days Total SB (hrs/day) = total leisure, care, meal and transportation time Results: Mean total SB (SD): 13.0 (4.6) hrs/day Mean total leisure sitting time: 7.5 (5.0) hrs/day Mean TV sitting time (SD): 2.3 (1.9) hrs/day Mean work sitting time (SD): 15.4 (14.0) hrs/week	Outcome type and measurement: HRQoL measured with Short Form-36 (SF-36) and Functional Assessment of Cancer Therapy – General (FACT-G) Results: No significant relationships found between total SB (hrs/day) and HRQoL ↑ TV watching correlated with ↓ scores on multiple QoL scales: functional well-being, social functioning, and overall physical composite score (PCS)* TV viewing time significantly predicted PCS*	Fair
Cárceles-Álvarez et al., 2020^34^ Spain	Cross-sectional	Age at diagnosis: < 15 yrs Age at enrollment: 8–18 yrs Treatment status: Off therapy	Examine the differences in HRQoL between survivors and a healthy comparison group Explore the clinical, environmental and lifestyle factors implicated in the HRQoL of survivors	Total N = 234 _________________ Survivors: n = 117 Mean age (SD): 13.2 yrs (3.0) Male: 53 (45%) Female: 64 (55%) _________________ Matched controls: *n* = 117	All extracranial cancer types Mean age at diagnosis (SD): 3.6 (2.9)	Off therapy: 100% Years since diagnosis: 5–10 yrs: 68 (58%) > 10 yrs: 49 (42%) ____________________ Treatment received: CT, RT or SG alone: 44 (38%) CT + SG: 33 (28%) CT + RT: 16 (14%) CT + BMT: 14 (12%) CT + RT + BMT: 10 (9%)	Measures: Self-report—Pediatric Environmental History (PEHis): screen time (hrs/day) Results: Survivors had higher screen time than controls 1–5 h/day: 70.9% survivors 61.4% controls ≥ 5 h/day: 17.9% survivors 9.6% controls	Outcome type and measurement: PedsQL 4.0 Generic Core Scales used to measure HRQoL Results: Screen time had small effect size (Cohens f^2^ < 0.15) on HRQoL Screen time ≥ 5 h/day associated with ↓ Emotional Functioning Subscale score* Screen time did not significantly modify other HRQoL scores	Fair
*Studies reporting on bone health*									
Kelly et al., 2009^40^ USA	Cross-sectional	Age at diagnosis: 3–18 yrs Age at enrollment: NR Treatment status: On and off therapy	Evaluate total body bone mass in children with ALL using pediatric-specific DXA algorithm and examine potential predictors of bone mass	Total N = 41 Median age: 10 yrs Range: 3–19 yrs Males: 25 (61%), Females: 16 (39%) White: 16 (39%) Hispanic: 21 (51%) _______________ Controls: NA	ALL Age at diagnosis (range): 3–18 yrs	On therapy: 21 (51%) Off therapy: 20 (49%) ____________________ CT: 100% Cranial RT: 9 (22%)	Measurement: Self-report – Modified Slemenda questionnaire: screen time (TV/computer) in hrs/week Results: Summary screen time data for survivors not reported	Outcome type and measurement: Total body bone mineral content (g), total body bone area (cm2), and total body areal BMD (g/cm2) Z-scores measured by DXA Results: Screen time (hrs/wk) not a significant predictor of bone measurement Z- scores	Fair
Gunes et al., 2010^39^ Turkey	Cross-sectional	Age at diagnosis: NR Age at enrollment: NR Treatment status: Off therapy	Determine growth and BMD, tendency to osteoporosis, and risk factors influencing BMD after treatment for childhood ALL	Total N = 70 Mean age (SD): 10.6 (3.8) yrs Males: 41 (59%) Females: 29 (41%) __________________ Controls: NA	ALL Age at diagnosis: Mean (SD): 5.7 (3.4) yrs Range: 1.2–14.5	Off therapy: 100% Mean time interval since therapy completion (SD): 45.5 (30) months Range: 1—97 months ____________________ CT: 100% Cranial RT: 29 (41%)	Measurement: Self-report – Question on hrs/day watching TV or on computer within the past 3 days: mean daily screen time, hrs/day Results: Mean screen time (SD): 3.5 (1.7) hrs/day	Outcome type and measurement: BMD measured via DXA Results: No significant association between screen time and BMD in multivariate analysis	Fair
Polgreen et al., 2012^43^ USA	Cross-sectional	Age at diagnosis: NR (≥ 5 yr from diagnosis) Age at enrollment: 9–18 yrs Treatment status: Off therapy	Determine prevalence and severity of bone deficits in CCS vs. sibling controls Identify modifiable lifestyle and treatment-related factors associated with bone deficits in CCS	Total N = 527 _________________ Survivors: n = 319 Mean (SE): 14.5 (0.1) yrs Males: 171 (54%) Females: 148 (46%) White/not Hispanic: 274 (89%) ________________ Controls: n = 208	All cancer types included Mean age at diagnosis (SD): 4.5 (0.2) yrs Range: 0—12.5 yrs	Off therapy: 100% Mean time since treatment (SD): 10.1 (0.2) yrs Range: 4.3—17.8 yrs ____________________ CT: NR RT: 74 (23%) Cranial RT: 31 (10%)	Measurement: Self-report – Modifiable Activity Questionnaire for Adolescents: TV/computer screen time (hrs/day) Results: CCS similarly sedentary to controls Screen time (n, %) 0–1 h/day: CCS: 73 (25%) Controls: 52 (26%) > 1 h/day: CCS: 225 (76%) Controls: 147 (74%)	Outcome type and measurement: BMD measured by DXA Results: ↑ screen time (≥ 2 h/day) associated with ↓ whole body BMD Z-score	Good
Marmol-Perez et al., 2023^41^ Spain	Cross-sectional	Age at diagnosis: NR (≥ 1 yr from diagnosis) Age at enrollment: 6–18 yrs Treatment status: Off therapy	Examine the associations of 24-h movement behaviors (MVPA, LPA, SB, and sleep) with areal BMD parameters in childhood cancer survivors	Total N = 116 Mean age: 12.1 (3.3) yrs Males: 58% Females: 42% _________________ Controls: NA	All cancer types	Off therapy: 100% Mean time from treatment completion (SD): 5.0 (3.8) yrs __________________ CT: NR RT: 28%	Measurement: Device—ActiGraph wGT3x-BT accelerometer (worn on wrist for 7 consecutive days): mean total ST (min/day) used to calculate geometric mean Results: Geometric mean for ST (min/day): ↑ SB in older peri/post-pubertal vs. prepubertal survivors Pre-pubertal: 556.0 min/d Peri/Post-pubertal: 697.5 min/d	Outcome type and measurement: aBMD (g/cm2) of total body and multiple local sites obtained via DXA Compositional data analysis performed to investigate time allocation across behaviors (SB, LPA, MVPA) Results: In pre- and post-pubertal survivors: ST → LPA: ↑ aBMD* MVPA and LPA → ST: ↓ aBMD*	Good
Marmol-Perez et al., 2024^42^ Spain	Cross-sectional (same population from 2023 study)	As above	Investigate association of TV watching time with bone parameters in survivors Examine if high lean mass attenuates negative association of TV watching > 1 h/day with bone parameter in survivors	As above	As above	As above	Measurement: Self-report – Adapted Youth Activity Profile (YAP) questionnaire: 7-day recall of TV watching time in hrs/day. Grouped as 0 h, < 1 h and > 1 h for analysis Results: 82% reported watching TV TV watching time - n (%): 0 h: 21 (18%) < 1 h: 44 (39%) ≥ 1 h: 49 (43%)	Outcome type and measurement: Multiple bone parameters, including aBMD (g/cm2), and lean mass measured via DXA Results: No significant associations in pre-pubertal survivors In peri/post-pubertal survivors: ↑ TV watching associated with multiple ↓ bone parameters* ↑ lean mass associated with ↑ bone parameters in those with TV watching ≥ 1 h*	Good
Gil-Cosano et al., 2024^38^ Spain	Cross-sectional	Age at diagnosis: NR (≥ 1 yr from diagnosis of childhood cancer) Age at enrollment: 6–18 yrs Treatment status: Off therapy	Investigate associations of PA and SB with markers of bone turnover and formation	Total N = 116 Mean (SD): 12.1 (3.3) yrs Male: 6 (58%) Female: 49 (42%) ________________ Controls: NA	All cancer types	Off therapy: 100% Mean time from treatment completion (SD): 5.0 (3.8) ____________________ CT: NR RT: 28 (39%)	Measurement: Device—ActiGraph wGT3x-BT accelerometer (worn on wrist for 7 consecutive days): mean total ST (min/day) Total PA and LPA also reported (min/day) Results: Mean total ST (SD): 624.9 (103) min/day	Outcome type and measurement: Bone turnover assessed with lab biomarkers of bone formation and resorption (CTX, PTH, P1NP, total OC, alkaline phosphatase) and calculation of biomarker uncoupling complexes Results: ↑ Total ST (min/day) associated with ↓ markers of bone formation and favored bone resorption ↑ LPA (min/day) favored bone formation	Fair
*Studies reporting on physical fitness*									
Malicka et al., 2020^44^ Poland	Cross-sectional	Age at diagnosis: NR Age at enrollment: NR Treatment status: Off therapy	Assess physical fitness and PA levels of children treated for leukemia Evaluate relationship between PA, SB, and physical fitness	Total *N* = 34 Mean age: 11.3 (2.8) yrs Males: 23 (68%) Females: 11 (32%) ________________ Controls: NA	ALL, AML	Off therapy: 100% Mean time since treatment completion: 3.09 (1.80) yrs ____________________ Treatment received: CT: 100% RT: 2 (6%) BMT: 8 (24%)	Measure: Self-report—Health Behavior in School-Aged children (HBSC) questionnaire: time watching television, time playing sedentary games, and time spent using a computer over the last 7 days (hrs/day for each domain) Results: % reporting ≥ 2 h daily of: Week day TV watching: 32% Weekend TV watching: 77% Week day video games: 50% Weekend day video games: 82% Week day electronic device use: 35% Weekend days using electronic devices: 61% Weekday screen time ↓ and overall video game time ↑ as time off therapy ↑	Outcome type and measurement: Physical efficiency/fitness via: Palant ball throw Long jump 60 m run Results: ↑ Weekday screen time (gaming and computer use) associated with ↓ performance in palant ball throw and long jump* No association found between screen time and run performance	Poor

^*^Indicates statistically significant finding

Abbreviations: *ALL* acute lymphoblastic leukemia, *AML* acute myeloid leukemia, *BMI* body mass index, *(a)BMD* (areal) bone mineral density, *CF* chronic fatigue, CNS central nervous system, *CCS* childhood cancer survivors, *CPET* Cardiopulmonary exercise test, *CRF* Cardiorespiratory fitness, *CSI* craniospinal irradiation, *CT* chemotherapy, *DXA* dual energy x-ray absorptiometry, *FB* Facebook, *IQR* interquartile range, *LPA* light physical activity, *MVPA* moderate-to-vigorous physical activity, *MPA* moderate physical activity, *NR* not reported, *PA* physical activity, *QoL* quality of life, *RT* radiation therapy, *SB* sedentary behavior, *SD* standard deviation, *SE* standard error, *SG* surgery, *ST* sedentary time, *TV* television, *USA* United States of America, *VPA* vigorous physical activity

Sample sizes ranged from 21 to 330 participants, totaling 1,690 cancer patients and survivors across all studies. Mean/median age of study participants overall ranged from 10 to 33.7 years. The number of included participants in each age/treatment category were: (1) child/adolescent cancer patients on therapy (*n* = 95) [14, 31, 36, 40]; (2) child/adolescent survivors of childhood/adolescent cancer (*n* = 937) [14, 33, 34, 38–44]; (3) YA cancer patients on therapy (*n* = 24) [35]; (4) YA/adult survivors of childhood/adolescent or YA cancer (*n* = 464) [30, 35, 37]. Additionally, one study enrolled 170 survivors of either childhood or YA cancer which included participants from 6–40 years old at time of study (mean age 19.4 years) [32].

Six studies measured SB via accelerometry, with wrist-worn wGT3x-BT accelerometers (ActiGraph LLC, Pensacola, FL, USA) being the most common device (*n* = 3) [30, 38, 41]; other devices utilized included activPAL (thigh-worn; PAL Technologies Ltd., Glasgow, UK; *n* = 1) [37], Actical (hip-worn; Philips Respironics, Bend, OR, USA; *n* = 1) [14], and Actiwatch 2 (wrist-worn; Philips Respironics, Bend, OR, USA; *n* = 1) [36]. The most common metric reported for device-measured SB was total sedentary time in minutes per day (*n* = 4) [14, 30, 37, 38]. Other reported device-measured metrics were sedentary bouts per day (*n* = 1) [14], percentage of the day spent sedentary (*n* = 1) [30], immobile time (*n* = 1) [36], and the geometric mean of sedentary time per day (*n* = 1) [41].

Ten studies measured SB via self-report alone using various surveys [31–35, 39, 40, 42–44]; only two studies used the same validated questionnaire [Health Behavior in School-Aged Children (HBSC)] [44, 48]. Screen time was the most commonly-studied self-report SB domain, including watching television (TV) and using a computer (*n* = 11) [30–35, 39, 40, 42–44], with other domains including time spent playing video games (*n* = 3) [31, 33, 44], total reported SB (*n* = 1) [35], and sitting time at leisure and work (*n* = 1) [35]. Five studies examined the association between SB and cardiometabolic outcomes [14, 30–33]; four investigated SB and PROs [34–37]; six evaluated SB and bone health [38–43]; and one reported on SB and physical/musculoskeletal fitness [44]. No included studies reported on SB associations with mortality.

#### Cardiometabolic Outcomes

Overall, greater SB was associated with lower cardiorespiratory fitness (CRF; *n* = 2), poorer body composition metrics (*n* = 2), and increased cardiovascular disease risk factors (CVDRF; *n* = 1). Braam et al. found that device-measured SB (total min/day) had a significant negative correlation with CRF in a mixed sample of child/adolescent patients and survivors [14]. Similarly, Mizrahi et al. found that self-reported hours/day of screen time showed a negative association with CRF in child/adolescent survivors of childhood/adolescent cancer, although this did not reach statistical significance (*p* = 0.067) [33]. However, a small study of patients being treated for childhood leukemia by Kowaluk and Woźniewski did not find an association between SB and CRF [31]. Large cross-sectional analyses showed that self-reported screen time was significantly associated with increased body and trunk fat [32], increased weight-to-height ratio (WtHR) [30], and decreased lean mass in YA/adult survivors of childhood cancer [30]. The one included prospective study reported that survivors who were sedentary for ≥ 60% of the day (measured via accelerometer) had increased risk of new onset high total cholesterol and any CVDRF [30].

#### Patient-reported outcomes

Overall, greater SB was associated with lower health-related quality of life (HRQoL) metrics (n = 2) [34, 35] and mixed evidence related to sleep metrics and fatigue (n = 2) [36, 37]. Both studies assessing HRQoL found that self-reported screen time, specifically total TV time and TV time ≥ 5 h/day, was associated with varied decreases in HRQoL scores in child/adolescent survivors of childhood/adolescent cancer and patients and survivors of YA cancer [34, 35]. One study examined the effect of SB on sleep and found that higher device-measured SB was associated with poorer sleep outcomes in child/adolescent cancer patients receiving therapy [36]. Zeller et al. were the only group to study SB and fatigue and did not find significant associations with device-measured SB in YA/adult survivors of childhood cancer [37].

#### Bone health outcomes

The most frequently-studied outcome was bone health, and all but one [40] of these studies included only child/adolescent survivors of childhood/adolescent cancer (n = 5) [38, 39, 41–43]. Dual energy x-ray absorptiometry (DXA) was used in five of the six studies to measure bone mineral density (BMD) and other bone parameters [39–43]. Overall, findings across studies were mixed, with two reporting no significant relationships [39, 40], one reporting a negative association [43], one reporting associations that differed based on pubertal status and lean mass [42], and two reporting associations that differed based on allocation of SB time in comparison to other activity behaviors [38, 41].

The two studies reporting no significant associations measured total self-reported screen time [39, 40] while the one study reporting a negative association found that the threshold ≥ 2 h/day of self-reported screen time was associated with a lower whole body BMD z-score [43]. Another study found no association in pre-pubertal survivors but noted a significant negative correlation in peri/post-pubertal survivors between self-reported TV time and multiple parameters of bone health in adjusted analyses, including total body and hip BMD that were attenuated with greater lean mass [42].

Marmol-Perez et al. utilized compositional data analysis to evaluate the effect of different time allocation across SB, LPA, and MVPA on BMD and found that replacing SB with LPA was associated with greater BMD in child/adolescent survivors of childhood/adolescent cancer [41]. Conversely, time allocated from MVPA and LPA to SB was associated with lower BMD [41]. Finally, Gil-Cosano et al. evaluated relationships between device-measured SB and blood biomarkers of bone turnover; they reported that greater device-measured SB was associated with decreased markers of bone formation and favored bone resorption in child/adolescent survivors [38].

#### Physical fitness outcomes

One study among 34 child/adolescent cancer survivors evaluated physical fitness as measured via palant ball throw (test of upper-body throwing strength), long jump, and 60 m run [44]. Greater self-reported weekday screen time was associated with decreased performance in the palant ball throw and long jump but not with run performance [44].

#### Quality assessment

Of the 16 observational studies included, overall quality was rated as Good for 7 studies [30, 32, 33, 36, 41–43]; Fair for 7 studies [14, 34, 35, 37–40]; and Poor for 2 studies [31, 44]. Fair ratings were typically due to limited control for confounding, insufficient reporting of sample size justification, and use of non-validated SB measures. The two studies rated as Poor received this classification due to unclear recruitment methods, limited methodological transparency, lack of control for confounding, and unclear reporting of study outcomes [31, 44].

### Intervention studies aiming to reduce SB

#### Study characteristics

Intervention studies are summarized in Table 2. Eight articles representing seven intervention studies were included [45–52]. Study designs included randomized controlled trials (n = 5) [47–51], an observational two-arm parallel group study (n = 1) [45], and a single-arm pilot trial with pre-post assessments (n = 1) [46]. Publication dates ranged from 2015 to 2023. The studies were conducted in the U.S. (n = 4) [45, 47, 49, 51], Australia (n = 1) [46], Poland (n = 1) [48], and Sweden (n = 1) [50].

**Table 2:** Details of included interventional studies

Author(s), year published, country	Study design	Inclusion criteria	Study aims	Sample size and characteristics	Cancer diagnoses	Treatment history	Intervention type & goals	Intervention duration	SB measure	Results
Gill et al., 2016^45^ USA	Observational two-arm parallel group study	Age at diagnosis: NR Age at enrollment: 18–39 yrs Treatment status: On and off therapy	Primary: Compare effects of outdoor therapy camp on PA vs. wait list control during camp and 3 months later Secondary: Examine effects on SB and correlates of PA	Total *N* = 116 Mean age (SD): 32.4 (6.5) yrs Female: 88 (79%) White: 96 (88%) ____________ Intervention: 50 Control: 66	All cancer types included __________ Intervention: Mean age at diagnosis (SD): 27.3 (6.0) yrs __________ Control: Mean age at diagnosis (SD): 30.1 (7.6) yrs	Intervention: In remission: 31 (62%) Relapsed: 16 (32%) CT: 43 (88%) RT: 32 (64%) ________________ Control group: In remission: 33 (60%) Relapsed: 10 (17%) CT: 45 (78%) RT: 29 (49%)	Two-arms: 1) Outdoor adventure camp × 1 week: 5–7 h/day of instruction 2) Waitlist control Goals: Teach mastery in outdoor adventure activity, ↑ PA, ↓ SB	7-day camp experience + 3-month follow-up (baseline 1 month before camp)	Self-report: 7-day physical activity recall (PAR)—2 questions on hrs/wk of watching TV and other sitting time (non-TV) PA: 7-day PAR—min/day of moderate, hard, and very hard intensities of PA; summed to calculate total PA in min/wk	*Within group difference*: Compared to baseline, camp group ↓TV watching by 7.1 h/wk vs control group ↑ 0.7 h/wk at 1 week* Compared to baseline, camp group ↓ other sitting time by 16.6 h/wk vs. control group ↓ 1 h/wk* No differences in TV watching or other sitting time between baseline and 3 months. Returned to baseline after camp Compared to baseline, camp group ↑ PA by 577 min/wk at 1 wk and by 133 min/wk at 3 mos vs. control group ↑ 9 min/wk and ↓ 75 min/wk*
Sabel et al., 2016^50^ Sweden	Two-arm crossover RCT	Age at diagnosis: NR Age at enrollment: 7–17 yrs Treatment status: 1–5 yrs off therapy	Explore effects of active video gaming (AVG) on energy expenditure (EE) levels during sessions and overall intervention Evaluate compliance and feasibility of the method	Total *N* = 13 ____________ Mean age (SD): 12.5 (2.9) yrs Male: 6 (46%) Female: 7 (54%) ___________ Intervention: 7 Waiting list: 6	All with CNS tumors	Off therapy: 100% _______________ RT: Focal: 3 (23%) CSI + focal: 10 (77%) CT: 12 (92%) Major neurosurgery: 9 (69%)	Two arms with planned crossover: 1) AVG intervention: used NintendoWii, goal AVG 30 min/day × 5 d/wk, video conference coaching weekly 2) Waitlist control Goals: ↑ PA, ↓ SB	10–12 weeks	Device: SenseWear Pro 2 Armband (Worn continuously for 1 week at baseline, and every second week during AVG period); min/day total ST PA: Device – Min/day MVPA	*Within group changes for all participants during intervention periods (crossover analysis not done*): No significant change in total ST (↓ 20 min/day) and MVPA (no change) compared to baseline
Mendoza et al., 2017^49^ USA	Two-arm RCT	Age at diagnosis: NR Age at enrollment: 14–18 yrs Treatment status: Off therapy, ≥ 1-year post-	Primary: Test feasibility of intervention Exploratory: Examine changes in PA, SB, motivation, and QoL	Total *N* = 59 ____________ Mean (SD): 16.6 (1.5) yrs Male: 24 (41%) Female: 35 (59%) Non-Hispanic White: 42 (71%) ____________ Intervention: 29 Control: 30	All cancer types	Off therapy: 100% Median duration off therapy: 10.3 years IQR: 7.5–13.0 ________________ Treatment hx: CT: 50 (85%) RT: 16 (27%) Cranial RT: 13 (22%) Leg/pelvis RT: 6 (10%)	Two-arms: 1) Fitbit + Facebook intervention: Weekly tailored goal setting for steps, badges and posts in Facebook weekly, affective text messages every other day 2) Usual care Goals: ↑ PA, ↑ steps, ↑ motivation	10 weeks	Device: ActiGraph GT3X + accelerometer (worn on the hip for 7 days continuously); min/day total ST PA: Device—min/day MVPA	*Between group differences (Baseline to 10 weeks):* No significant difference in total ST mean change between groups (intervention ↓ ST 4.5 min/day) No significant difference in MVPA mean change between groups (intervention ↑ MVPA 4.4 min/day)
Ha et al., 2022^46^ Australia	Single-arm pilot intervention (pre-post design)	Age at diagnosis: NR Age at enrollment: 8–13 yrs Treatment status: Off therapy, ≥ 12 mos post-	Primary: Evaluate feasibility and acceptability of intervention Secondary: Assess changes in PA levels and behaviors, aerobic fitness, and (HRQoL)	Total *N* = 27 (at baseline) _____________ Received intervention: *n* = 23 (4 withdrawn) ____________ Mean age (SD): 10 (1.5) yrs Female: 12 (44%)	All cancer types included	Off therapy: 100% Mean time since treatment completion (SD): 5 (3.1) yrs _________________ Treatment received: CT: 26 (96%) SG: 17 (63%) RT: 4 (15%) BMT: 4 (15%)	Single-arm – iBounce intervention: a) Adapted digital health education program with digital app: 10 education modules, completed 1-2x/week b) App-integrated wearable activity trackers: sync weekly with app Goals: ↑ PA, improve PA behaviors	12 week intervention + 24 week follow-up	Device: GENEActiv accelerometer (worn on wrist for 24 h × 7 days); hrs/day total ST in bouts ≥ 60 s and ≥ 300 s PA: Device—Mins/day MVPA in bouts ≥ 3 s	*Within group difference*: Compared to baseline, no change in ST (bouts ≥ 60 s) at 12 weeks (5.6 h/day pre- and post-) Compared to baseline, no change in MVPA at 12 weeks (↓ 2.5 min/d) 3/11 (27%) with complete data at 24 weeks moved to more active cluster group (of 5 defined groups, most to least active) based on PA and SB levels
Johnson et al., 2022^47^ USA	Two-arm pilot RCT	Age at diagnosis: NR (1–6 yrs from diagnosis) Age at enrollment: 18–39 yrs Treatment status: Off therapy, ≥ 12 mos post-	Primary: Test feasibility of intervention among young adult cancer survivors Exploratory: Examine changes in ST, MVPA, QoL, and self-determination theory constructs	Total *N* = 49 _____________ Mean age (SD): 33.6 (4.9) yrs Male: 21 (43%) Female: 27 (55%) Other: 1 (2%) Non-Hispanic White: 31 (63%) ____________ Intervention: 26 Control: 23	All cancer types included Mean age at diagnosis (SD): 30.0 (5.1) yrs	Off therapy: 100% Mean years since diagnosis (SD): 3.6 (1.2) yrs _______________ Surgery at primary site: 35 (71%) CT: 40 (82%) RT: 26 (53%)	Two arms: 1) Fitbit + Facebook intervention: Weekly tailored goal-setting for steps, 4x/week badges or posts in Facebook, PA “buddy”, motivational texts every other day 2) Fitbit only control Goals: ↑ steps, ↑ PA, ↓ ST	12 weeks	Device: ActiGraph GT3X + accelerometer (worn on the hip for 7 days continuously); min/day total ST PA: Device—Min/day LPA & MVPA	*Between group differences (Baseline to 12 weeks)*: Compared to baseline, intervention group ↓ ST by 52.4 min/day vs. control group ↑ by 2.5 min/day* (*adjusted*) No change in MVPA (mean change 0) and LPA ↓ 21.2 min/day (ns) from baseline to post-intervention
Kowaluk & Woźniewski, 2022^48^ Poland	Two-arm RCT	Age at diagnosis: NR (**≤ **6 mos from diagnosis) Age at enrollment: 7–13 yrs Treatment status: On therapy	Primary: Evaluate effect and feasibility of interactive video game (IVG) program in children undergoing leukemia treatment Secondary: Assess effects on CRF, PA, and SB	Total *N* = 21 _____________ Male: 12 (57%) Female: 9 (43%) ____________ Intervention: 10 Control: 11	ALL: 13 (62%) AML: 8 (38%)	On therapy: 100% Mean treatment duration (SD): 6.2 (1.6) mos ________________ CT: 100%	Two-arms: 1) IVG program: IVG sessions 3x/week × 4 weeks (12 total sessions), 4 IVGs/session with ↑ intensity 2) Usual care Goals: ↑ PA, ↓ SB	4-week intervention + 14 mo follow-up	Self-report: Health Behavior in School-Aged children (HBSC 2018) questionnaire – 4 questions on time in front of screens, playing sedentary games, and using the Internet over the last 7 days (hrs/day) PA: HBSC—days/wk with MVPA ≥ 60 min, frequency of VPA	*Between group differences* (*Baseline to 4 weeks and 14 months*): No significant difference in SB levels in any domain (screen time, video games, internet) between intervention and control at 4 weeks or 14 months Intervention group ↑ MVPA at 4 weeks vs. control (change in frequency not reported). No difference at 14 months *Within group differences* (*Baseline to 4 weeks and 14 months*): IVG group ↓ SB in all three domains at 4 weeks and 14 months (change in hrs/day not reported)
Valle et al., 2023^51^ USA	Two-arm, single-blinded, parallel group RCT	Age at diagnosis: 18–39 yrs Age at enrollment: 18–39 yrs Treatment status: Off therapy	Primary: Evaluate efficacy of intervention on total PA over 12 months Secondary: Assess changes in MVPA, LPA, SB and the proportion meeting PA guidelines	Total *N* = 280 ___________ Mean age (SD): 33.4 (4.8) yrs Male: 51 (18%) Female: 229 (82%) Non-Hispanic White: 215 (77%) ___________ Intervention: 140 Control: 140	All cancer types included	Off therapy: 100% Mean time from diagnosis (SD): 3.7 (2.4) yrs _______________ Treatment: CT: 172 (61%) SG: 242 (86%) RT: 128 (46%) BMT: 23 (8%) Hormone therapy: 14 (5%) Immunotherapy: 12 (4%)	Two-arm: 1) IMPACT intervention (Digital Tools Plus): Digital tools as below + PA-focused FB posts 5x/wk, adaptive website with behavioral lessons and tailored PA feedback 1x/wk, tailored texts 5x/wk 2) Self-help control (Digital Tools): Fitbit, smart scale, arm-specific FB group, and kick-off video chat session Goals: ↑ total PA, ↑ MVPA, ↑ steps, ↓ SB	12 months with ↓ support during months 7–12	Device: ActiGraph GT3X + accelerometer (worn on the wrist for 7 days continuously); min/day total ST: Device PA measures: min/wk total PA, MVPA, LPA, and steps/day Self-report SB (secondary): Sedentary Behavior Questionnaire – Total min/day SB, summed from 8 SB domains, both weekday and weekend Self-report PA (secondary): Godin Leisure Time questionnaire – frequency and duration (min/wk) of LPA, MPA, and VPA per week	*Between group differences (baseline to 6 months):* Device measured: Mean change in all outcomes (total ST, total PA, MVPA, LPA and steps) between groups not significant at 6 months Self-report: Mean change in all outcomes (total ST, total PA, MVPA, LPA) between groups not significant at 6 months *Within group differences (baseline to 6 months):* Device measured: Mean change in SB, total PA, and LPA within intervention group not significant at 6 months MVPA ↑ by 24.7 min/wk in intervention group and ↑ by 11.4 min/wk in control group at 6 mos* Steps ↑ by 670.7/day from baseline to 6 months among intervention group* Self-report (min/day): Mean change in SB within intervention group not significant at 6 mos Total PA ↑ by 123.3 min/wk in intervention group and ↑ by 83.4 min/wk in control group at 6 mos* MVPA ↑ by 77 min/wk in intervention group and ↑ 71.7 in control group at 6 mos* LPA ↑ by 33.8 min/wk in intervention group at 6 mos*
Valle et al., 2023^52^ USA	As above (article reported 12-month outcomes from same trial)	As above	Primary: Evaluate the efficacy of intervention on total PA over 12 months Secondary: Assess changes in MVPA, LPA, steps, SB and the proportion meeting PA guidelines Exploratory: Examine maintenance of PA from 6 to 12 months and identify potential moderators of intervention effects	As above 12 mo accelerometer data: 236 12 mo questionnaire data: 246	As above	As above	Intervention changes between 7–12 months: a) Website behavioral lessons ↓ to 1/mo in months 8, 10, and 12 b) Website tailored PA feedback ↓ to 1/mo in months 8, 10, and 12 c) Tailored text messages ↓ 1x/wk Continued with weekly adaptive PA goals and FB posts up to 5x/wk	As above	As above	*Between group differences (baseline to 12 months):* Device measured: Mean change in all outcomes (SB, total PA, MVPA, LPA and steps) between groups not significant at 12 months Self-report: Mean change in SB, MVPA and LPA between groups not significant at 12 months Mean total PA ↑ by 55.8 min/wk in intervention group vs. control group at 12 mos* *Within group differences:* Device measured: Mean change in SB, total PA, LPA and steps within intervention group not significant from baseline to 12 months ↑ in SB by 12.9 min/wk and ↓ in steps by 515.4 steps/day among intervention group between 6–12 mos* MVPA ↑ by 22.5 min/wk in intervention group and ↑ by 13.9 min/wk in control group from baseline to 12 months* Self-report: Mean change in SB within intervention group not significant from baseline to 12 mos Total PA ↑ by 125.7 min/wk in intervention group and ↑ by 69.6 min/wk in control group from baseline to 12 mos* MVPA ↑ by 88.6 min/wk in intervention group and ↑ 58.7 min/wk in control group from baseline to 12 mos* LPA ↑ by 36.8 min/wk in intervention group from baseline to 12 mos*

^*^indicates statistically significant result

Abbreviations: *ALL* acute lymphoblastic leukemia, *AML* acute myeloid leukemia, CNS – central nervous system, *CSI* craniospinal irradiation, *CT* chemotherapy, *CCS* childhood cancer survivors, *FB* Facebook, *IQR* interquartile range, *LPA* light physical activity, *MVPA* moderate-to-vigorous physical activity, *MPA* moderate physical activity, *PA* physical activity, *QoL* quality of life, *RT* radiation therapy, *SB* sedentary behavior, *SD* standard deviation, *SG* surgery, *USA* United States of America, *VPA* vigorous physical activity, *YACS* young adult cancer survivors

Sample sizes ranged from 13 to 280, with five studies having sample sizes < 60 participants [46–50]. Overall, a total of 565 patients and survivors were included. The mean age of participants ranged from 10 to 33.6 years. The number of reported participants (some baseline data missing [45]) in each age/treatment category were: (1) child/adolescent cancer patients on therapy (n = 21) [48]; (2) child/adolescent survivors of childhood/adolescent cancer (n = 99) [46, 49, 50]; (3) YA cancer patients on therapy (n = 26) [45]; (4) YA/adult survivors of childhood/adolescent or YA cancer (n = 393) [45, 47, 51, 52].

Five studies measured SB via accelerometry [46, 47, 49–51], with wrist (n = 1) [51] or hip (n = 2) [47, 49] worn ActiGraph GTX3 + accelerometer (ActiGraph LLC, Pensacola, FL, USA) being the most commonly used device. Two studies measured SB via self-report; one using 7-day physical activity recall (PAR) [45] and the other the HBSC questionnaire [48]. None of the included interventions had a primary aim to reduce SB, and the effects on SB were only measured as secondary or exploratory outcomes.

#### Intervention characteristics

Intervention durations ranged from 1 week to 12 months; of these, 57% (n = 4) were 10–12 weeks [46, 47, 49, 50]. Four studies tested a wearable fitness tracker with associated app and interactive websites [46, 47, 49, 51]. Three of these included arm-specific Facebook groups monitored by study staff as well as tailored goal setting and motivational text messages [47, 49, 51]. One study also included an optional “PA buddy” chosen by the participant [47]. Two studies utilized active video games [48, 50] and the remaining study tested the effect of outdoor adventure camp [45]. All had a primary focus on increasing PA and did not incorporate SB-specific goals for participants.

#### Intervention effects

Five studies primarily assessed intervention feasibility and acceptability and found that the interventions were generally feasible and well tolerated [46–50]. However, participant engagement with online components, such as social media groups and websites, varied across studies [46, 47, 49, 51, 52]. Retention rates were > 70% across most studies (n = 7) [45, 47–52], although completion of final assessments fell to 65–66% in two studies [45, 46]. Overall, intervention adherence was high among participants, ranging from approximately 70%−100% across included studies.

Overall, there was limited evidence for efficacy. Two studies [45, 48] reported significant decreases in SB within the intervention group, and one study [47] reported a significant decrease in SB among intervention participants versus controls. However, all observed effects were of short duration. Studies reporting reduced SB had varying intervention components, with only one employing a wearable fitness tracker [47] and two using interactive applications/websites [47, 48].

Gill et al. observed a significant decrease in self-reported sitting time (–16.6 h/week) and TV viewing (–7.1 h/week) immediately following a one-week outdoor camp among patients and survivors of YA cancer, although these effects were not sustained at 3-month follow-up [45]. Johnson et al. demonstrated a significant between-group reduction in device-measured SB (–52.4 min/day) over 12 weeks in a Fitbit + Facebook intervention group compared to a Fitbit-only control among survivors of YA cancer [47]. An interactive video game–based intervention led to within-group reductions in screen-based behaviors and increased physical activity (PA) among children undergoing leukemia treatment, although between-group differences were not statistically significant [48].

In contrast, the remaining studies reported no significant within-group or between-group differences in SB [46, 49–52]. The largest trial (n = 280) with the longest intervention period (12 months), conducted by Valle et al., observed no significant intervention effects on sedentary time at 6 or 12 months among survivors of YA cancer using both device-based and self-report measures, although there were small within-group improvements in PA [51, 52].

#### Risk of bias assessment

**Fig. 2:**
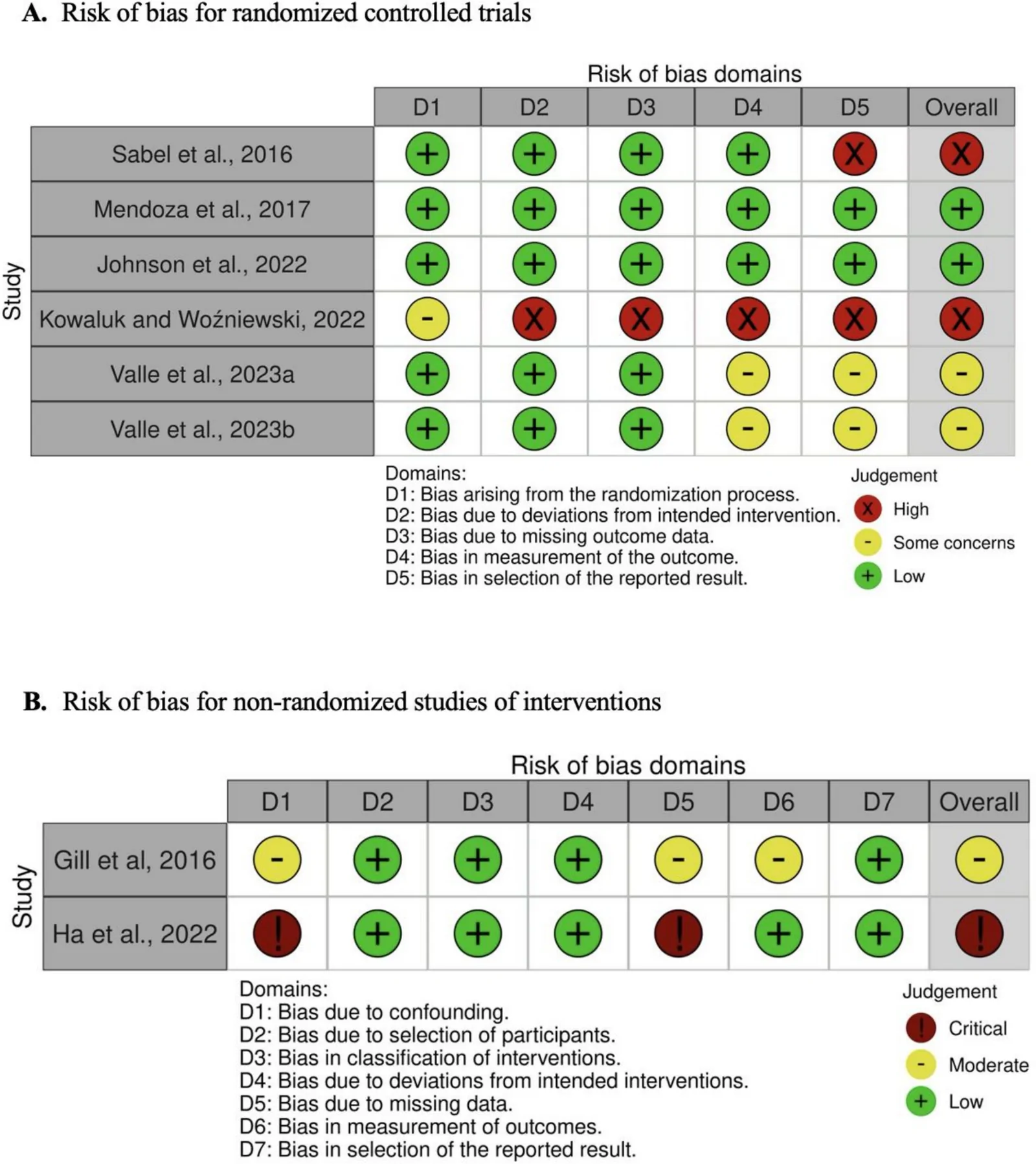
Risk of bias assessment for included intervention studies

Risk of bias assessments for intervention studies are shown in Fig. 2. Six RCT articles were assessed with the ROB2 tool (results for one study reported in two articles) [47–52]. Of these, two had some concerns due to risk of social desirability bias with self-report SB measures and limited information on SB analysis in the study protocol [51, 52]. Two had high risk of bias due to lack of information about missing data, lack of specification of and/or adherence to the pre-determined analysis plan, and concern for selected reporting of results from multiple analyses [48, 50]. Two non-randomized studies were assessed with the ROBINS-I tool [45, 46]. One was judged to be at moderate risk of bias due to lack of control for confounding factors and absence of a clearly pre-specified analytic approach [45]. The second non-randomized study was rated at critical risk of bias given lack of control for any confounding factors and a significant amount of missing data [46].

## Discussion

This scoping review summarizes existing literature on health outcomes and interventions related to SB in childhood and AYA cancer patients and survivors. To our knowledge, this is the first review to evaluate the clinical impact of SB in this population. Overall, our findings indicate there is a lack of published studies in the area, particularly prospective observational studies and intervention studies specifically targeting SB reduction. Preliminary evidence suggests that higher levels of SB are associated with select adverse health outcomes. However, intervention studies to date have demonstrated limited effectiveness in reducing SB.

Of the observational studies identified, all but two [30, 37] used a cross-sectional design [14, 32–39, 39–44, 48], which limits causal inference but provides early, hypothesis-generating evidence. The most commonly examined health outcomes were cardiometabolic factors (*n* = 5) [14, 30, 32, 33, 48] and bone health (*n* = 6) [38–43]. Higher SB was associated with lower CRF [14, 33], higher fat mass [30, 32], lower lean mass [30, 32], higher risk of onset of CVDRF [30], and lower BMD [41–43], consistent with findings in healthy children [10, 11, 54–57]. Limited evidence also suggests a negative effect of SB on HRQoL and sleep [34–36], though data remain inconclusive.

These findings are particularly important because childhood and AYA cancer patients are at higher risk for CVD, low CRF, low BMD, and reduced HRQoL due to treatment effects [58–60] and face decades of life as survivors. Evidence from healthy adult and pediatric populations suggests that reducing SB may improve these outcomes [61–64], potentially mitigating treatment-related toxicity and lowering risk of morbidity and mortality. However, epidemiologic research in childhood and AYA cancer survivors is hindered by a lack of large prospective studies and standardized SB measures, limiting cross-study comparisons and the development of evidence-based guidance. Based on our findings and given the overall infrequency of childhood and AYA cancer, the approach most likely to generate robust evidence regarding SB is through national/international consortia, working groups, and data repositories. Similar to the large-scale datasets of molecular findings being created from patients on clinical trials, we recommend that health behavior profiles, including standardized SB assessments, be incorporated for clinical trial participants to allow for large-scale data collection and comparison to existing data in healthy populations to inform future SB guidelines for this population.

None of the intervention studies targeted SB as a primary aim, and most focused on promoting PA alone [45–52]; thus, limited effects on SB are not unexpected. Three studies did demonstrate modest, short-term reductions in sedentary time [45, 47, 48], but no consistent intervention characteristics associated with SB reduction emerged to guide future strategies. Because SB is a distinct behavior from PA, it requires different behavioral intervention approaches [13, 65]; yet no included studies specifically targeted SB. This is a critical research gap for childhood and AYA cancer patients and survivors, particularly as prior reviews indicate that SB-specific interventions may be more effective in reducing SB than PA-focused or combined approaches in both general and clinical populations [66, 67]. Evidence from healthy AYA populations illustrates that SB-specific interventions targeting the physical environment (e.g., sit–stand desks), social environment (e.g., family-based approaches, peer or group programs), and individual behavior (e.g., self-monitoring, goal setting) have successfully reduced SB, offering potential models for adaptation in oncology settings [68, 69]. However, adapting these strategies for youth with cancer presents unique challenges due to treatment-related symptoms and late effects such as pain and fatigue.

Noting that SB comprises up to 80% of waking hours during upfront therapy, our findings highlight growing evidence of its adverse effects in childhood and AYA cancer patients, underscoring the need for effective, targeted interventions introduced early in therapy [14, 70, 71]. Notably, interventions focused on interrupting prolonged sitting and replacing SB with LPA offer a potentially more feasible and acceptable strategy to improve lifestyle behaviors for patients receiving therapy, as opposed to those focused on MVPA. Early intervention potentially mitigates establishment of long-term sedentary lifestyles and may build self-efficacy for ongoing behavior change, including addition of exercise, later in therapy or after treatment. Finally, intervention during therapy could improve short-term outcomes and mitigate treatment-related side effects.

The results of this review must be evaluated in the context of its limitations. As a scoping review, we did not conduct meta-analysis or pooled synthesis of outcomes. Most included epidemiologic studies were small and cross-sectional in nature, limiting causal inference, and the limited number of prospective or longitudinal studies hampers our ability to assess the long-term effects of SB on health outcomes. In addition, heterogeneity in SB measurement, particularly among self-report measures, limits comparability across studies, and outcome measures varied widely, particularly among PROs and fitness assessments. Finally, few intervention studies were adequately powered or rigorously designed to detect changes in SB as a primary outcome.

## Conclusion

This scoping review highlights the emerging but still limited body of evidence on the health impacts of SB in childhood and AYA cancer patients and survivors. Included observational studies link SB to adverse cardiometabolic, skeletal, and psychosocial outcomes, while intervention studies show only modest SB reductions without targeted strategies. Longitudinal studies with standardized SB assessment and high-quality, SB-focused interventions are needed to clarify clinical impact and evaluate SB reduction as a viable strategy to improve health outcomes in young people affected by cancer.

## Supplementary Information

Below is the link to the electronic supplementary material.

Supplementary file1 (DOCX 16.7 KB)

## Data Availability

Data produced from this scoping review will be made publicly available via the study registration page in OSF ([https://osf.io/7apyv] (https:/osf.io/7apyv)) within 3 months of manuscript publication.
