# Supplementary material for: Sedentary behavior in children, adolescents, and young adults treated for cancer: A scoping review of health outcomes and interventions
Source: J Cancer Surviv. 2025 Nov 11;20(5):2685–709. doi: 10.1007/s11764-025-01908-z (PMC13723577; doi:10.1007/s11764-025-01908-z)
Supplement: Supplementary file 1 [file 11764_2025_1908_MOESM1_ESM.docx]

| **Supplemental Table 1** Search strategy for PubMed | |
| --- | --- |
| **Domain/Concept** | **Search Terms*** |
| Pediatrics | “Child”[MeSH] OR “Adolescent”[MeSH] OR “Young Adult” [MeSH] OR "Minors" [MeSH] OR "Pediatrics" [MeSH] OR "Hospitals, Pediatric"[MeSH] OR “paediatric*” OR “toddler*” OR “preschool*” OR “pre school*” OR “child*” OR “kid” OR “kids” OR “juvenile*” OR “minor*” OR “youth*” OR “youngster*” OR “girl*” OR “boys” OR “boys” OR “elementary school*” OR “grade school*” OR “preadolescen*“ OR “pre adolescen*” OR “preteen*” OR “pre teen*” OR “middle school*” OR “adolescen*” OR “teen*” OR “high school*” OR “pediatric*” OR “young adult*” |
| Cancer Patients & Survivors | “Neoplasms” [MeSH] OR “Cancer Survivors” [MeSH] OR (“Neoplasms” [MeSH] AND “Survivors” [MeSH]) OR “oncolog*” OR “neoplas*”OR “tumor*” OR “tumour*” OR “cancer*” OR “malignan*” OR “metasta*” OR “carcinogenesis” OR “carcinoma*” OR “sarcoma*” OR “leukemia*” OR “leukaemia*” OR “lymphoma*” OR “Hodgkin* disease” OR “glioma*” OR "astrocytoma*" OR “craniopharyngioma*” OR "ependymoma*” OR "medulloblastoma*” OR “retinoblastoma*” OR “neuroblastoma*” OR "Langerhans cell histiocytosis” OR “hepatoblastoma" OR "cholangiocarcinoma" OR “nephroblastoma” OR "rhabdomyosarcoma*” OR "osteosarcoma*” OR "fibrosarcoma*" OR “melanoma*” OR "angiosarcoma*" OR “antineoplastic*” OR “anti-neoplastic*” OR “anti neoplastic*” OR “cancer survivor*” |
| Sedentary Behavior | “Sedentary Behavior” [MeSH] OR “Sitting Position” [MeSH] OR “sedentary” OR “sedentary behaviour*” OR “sedentary behavior*” OR “sedentary lifestyle*” OR “sedentarism” OR “sedentary time” OR “sitting” OR “inactiv* lifestyle*” OR "inactiv* behavi*" OR “lack of physical activit*” OR “physical inactive*” OR “television” OR “television viewing” OR “television time” OR “television watching” OR “TV viewing” OR “TV time” OR “TV viewing” OR “screen time” OR “computer use” OR “computer time” OR “video gam*” |
| *Search terms, such as use of MeSH terms, are based on search in PubMed and have been adapted for each database as appropriate (Embase, Web of Science, CINAHL, and SportDiscus). | |
